# Perianastomotic pH Monitoring for Early Detection of Anastomotic Leaks in Gastrointestinal Surgery: A Systematic Review of the Literature

**DOI:** 10.1177/15533506241313168

**Published:** 2025-01-07

**Authors:** Josephine Walshaw, Katherine Hugh, Jack Helliwell, Joshua Burke, David Jayne

**Affiliations:** 1 Leeds Institute of Medical Research, St James’s University Hospital, 4468 University of Leeds, Leeds, UK

**Keywords:** anastomotic leak, gastrointestinal surgery, pH monitoring

## Abstract

**Introduction:**

Anastomotic leak (AL) represents a significant complication following gastrointestinal (GI) surgery, contributing to increased morbidity and mortality. pH monitoring has emerged as a potential diagnostic tool for the early detection of AL, but its effectiveness and clinical utility remain to be fully elucidated. This review aims to summarise the evidence regarding perianastomotic pH monitoring for AL detection.

**Methods:**

A systematic search of relevant databases was conducted to identify pre-clinical and clinical studies investigating pH monitoring for AL detection following GI surgery. Studies were screened by two independent reviewers based on predefined inclusion and exclusion criteria. Data were extracted and presented as a narrative synthesis.

**Results:**

A total of 10 studies were included in the review, comprising animal studies (n = 2), and human studies in upper GI (n = 3) and colorectal (n = 5) patients. Consistent findings of lower pH values in patients with AL across various postoperative time points were demonstrated. There was diversity in the pH detection method, in addition to variable frequency and timing of pH monitoring. Four studies reported a shorter time for AL detection with pH monitoring vs conventional methods, although no statistical comparisons were used. No standard pH cut-off value for AL detection was identified.

**Conclusion:**

pH monitoring shows potential as a diagnostic tool for the early detection of AL following GI surgery. While the existing evidence supports its potential utility, further research is required to establish standardised protocols and assess its clinical impact.

## Introduction

Anastomotic leakage (AL) represents a life-threatening complication following gastrointestinal (GI) surgery. The clinical consequences include abscess formation, faecal peritonitis, sepsis, and multiorgan failure, resulting in prolonged hospital stay and increased morbidity and mortality.^1,2^ If it occurs following oncological resection, there is a greater risk of local recurrence and reduced disease-free survival.^3^ The early diagnosis and effective management of AL are crucial in minimising adverse outcomes. There is a direct link between the timing of intervention and the severity of septic complications,^4,5^ with a delay in re-operation or definitive treatment escalating mortality rates from 24% to 39%.^6^

AL may manifest in various ways. While some cases present suddenly with fulminant sepsis and multi-organ failure, necessitating immediate surgical intervention, others may exhibit a more insidious presentation with ileus or failure to progress.^7^ In certain instances, leaks may remain subclinical with no apparent symptoms. In such scenarios, conventional diagnostic tests may fall short in detecting AL promptly, thereby hindering timely intervention and potentially increasing serious morbidity and mortality.

In this context, a promising approach involves the use of biomarkers from perianastomotic fluid for the early detection of AL.^8^ The tissue hypoxia and ischaemia that accompanies Al produces an acidic perianastomotic environment, that other factors, such as bacterial proliferation and inflammation, might contribute to.^9-12^ Therefore, monitoring the pH of the perianastomotic environment might enable AL to be detected earlier.

The aim of this review was to explore the literature regarding approaches to perianastomotic pH monitoring for the early detection of AL following GI surgery, including the timing and frequency of pH measurements, and pH levels associated with AL.

## Methods

### Protocol and Registration

The protocol for this review was prospectively registered with Prospero (Registration ID CRD42024500573).^13^ A systematic review of the literature was performed in accordance with the Cochrane Handbook for Systematic Reviews of Interventions^14^ and has been reported following the Preferred Reporting Items for Systematic Reviews and Meta-Analyses (PRISMA) guidelines.^15^

### Eligibility Criteria

All studies that described perianastomotic pH monitoring for AL detection following GI surgery in (i) adult patients over the age of 18, or (ii) animal models, were eligible for inclusion. Original clinical and pre-clinical research articles were included. Case reports, editorials, reviews, conference abstracts without available full text, and non-English language articles were excluded. Studies which assessed other biomarkers for AL detection were considered for inclusion, providing relevant data on pH was extractable.

For this study, pH was defined as the logarithmic measurement scale of hydrogen ion concentration. Surrogate markers of pH such as electrical conductivity or impedance were not included.

### Information Sources

A comprehensive systematic search was conducted from inception to 30th November 2023 using MEDLINE, EMBASE, and The Cochrane Library on 5th December 2023. Additionally, the ClinicalTrials.gov Website was searched for ongoing studies and authors were contacted for results. The reference lists of all included studies and screened full texts were manually reviewed for additional relevant papers. When full texts were not obtainable via conventional access methods, the authors and publishing journals were approached to request the full article text.

### Search Strategy

The search strategy was designed using relevant keywords, including (gastr* OR colo* OR “oesophag*”) AND (anastomo*) AND (heal* OR leak* OR fail*) AND (“pH” or acid*) (Appendix 1).

### Selection and Data Collection Process

Search results were uploaded onto the Covidence systematic review software,^16^ and duplicates were removed. Two independent reviewers (JW and KH) screened titles and abstracts for eligibility, and full text for potentially relevant articles for inclusion. Two independent reviewers (JW and KH) performed data extraction using a bespoke Excel data extraction spreadsheet. Any disagreement between reviewers at either stage was resolved through consensus or with a third reviewer (JB).

### Data Items

Extracted data included: (i) study characteristics including year of publication, country, study design, sample size, and setting, (ii) patient demographics including sex, age, and co-morbidities, (iii) intervention details including operation(s) performed, method of pH detection, and frequency of pH monitoring, and (iv) AL detection including pH value, interval from index procedure to AL detection, method for confirming AL, and diagnostic test accuracy data.

### Risk of Bias

Due to the heterogeneity of included studies, the use of a standard scoring tool for risk of bias was deemed inappropriate and therefore a formal risk of bias analysis was not performed.

### Synthesis Methods

Extracted data items were tabulated and a narrative synthesis approach was conducted in line with the Guidance on the Conduct of Narrative Synthesis in Systematic Reviews.^17^ This approach facilitated a structured analysis of a broad range of studies. Continuous data are presented as medians and interquartile ranges, or means and standard deviation. Categorical data are presented as frequencies and percentages. Where diagnostic 2 × 2 table data is provided (ie, true positives, true negatives, false positives, false negatives), this will be used to calculate diagnostic test accuracy outcomes, including those not reported directly in the original paper.

## Results

**Figure 1.**
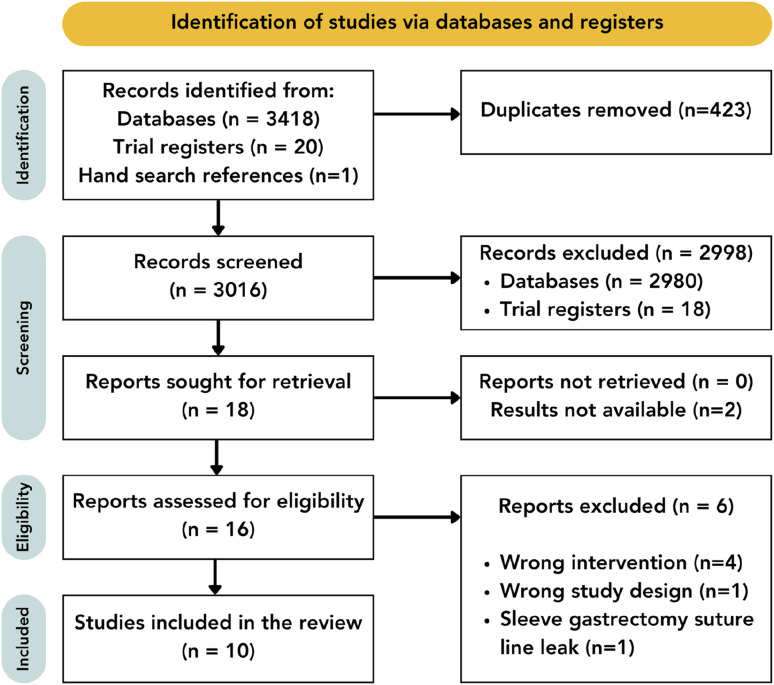
Preferred reporting items for systematic reviews and meta-analyses extension for scoping reviews (PRISMA) flowchart. n = number.

A total of 3016 articles were identified for title and abstract screening following duplication removal. Following screening, 18 reports were sought for retrieval with the results not yet available for 2 studies. The full texts of 15 studies were reviewed and, based on the inclusion and exclusion criteria, 6 studies were further excluded. An additional study was identified by hand-searching the references of the screened full texts, leading to 10 studies included in the final review (Figure 1).

### Study Characteristics

The 10 included articles encompassed two animal studies^18,19^ and eight human studies; grouped into five colorectal surgery^20-24^ and three upper GI surgery studies.^25-27^ The individual study results are summarised in Table 1.

**Table 1.** Study Characteristics Grouped Into Animal Studies, Upper Gastrointestinal Surgery and Colorectal Surgery.

First author	Year	Origin	Design	Number of participants (n)	Operation(s) performed and indication	Sex (M: F)	Age (median/mean years, range)	Number of participants with anastomotic leak (n, %)	Main study findings
Animal Studies									
Huynh et al^18^	2023	Canada	In vivo models (pigs)	2 pigs	P1 - Open abdomen leak (gastrotomy)	–	–	–	Sensors were able to detect the local changes in drainage pH within 10 min of leak induction
					P2 - Closed abdomen leak (needle aspiration of gastric fluid followed by open leak)				
Senagore et al^19^	1990	USA	In vivo models (pigs)	35 pigs	Colorectal resection at the sacral promontory with division of mesentery 10 cm proximal and distal to induce ischaemic insult	–	–	–	Intramural pH <7.0 predicted subsequent anastomotic outcome in 93%
Human Studies - Upper Gastrointestinal									
Linder et al^25^	2017	Sweden	Prospective cohort	32	Oesophagectomy for oesophageal cancer (adenocarcinoma n = 26, squamous cell carcinoma n = 6)	28:4	Median 64.5	6 (18.6%) - 2 minor, 4 major	Each surgical step altering the vascular supply to the gastric conduit resulted in detectable changes in pH. Patients with low pH on POD1 were more prone to have clinically relevant AL
Tarui et al^26^	1999	Japan	Prospective cohort	39	Oesophagectomy for oesophagal cancer	31:8	Mean 58.2 (44-73)	13 (33.3%) - 6 minor, 7 major	Significant difference in corrected pH between patients who experienced AL and those who did not from the morning of POD 1 until the morning of POD 2 (*P* < 0.05)
Machens et al^27^	1996	Germany	Prospective cohort	12	Oesophagectomy (oesophageal cancer of middle and distal third n = 12, brochial carcinoma infiltrating the oesophagus n = 1, oesophageal perforation following bouginage for achalasia n = 1)	Not stated	Not stated	6 (50%) - 2 minor, 4 major	Median pH in AL was lower than controls immediately following surgery and the following 5 days. Intramural pH 7.2 on POD 2 was 80% sensitive and 67% specific for AL
Human Studies - Colorectal									
Ge et al^20^	2021	China	Prospective cohort	300	Rectal resection for rectal cancer	170:130	Mean 65.8 (23-89)	21 (7.0%)	There was no difference in pH value between the AL and non-AL groups
Molinari et al^21^	2019	Italy	Prospective cohort	173	Right hemicolectomy, left hemicolectomy, or lower anterior resection (colorectal cancer n = 115, diverticulosis n = 18)	112:61	Median 72 (25-89)	16 (9.2%)	pH <7.32 on POD 1 and pH <7.21 on POD 3 were independent risk factors for AL. Using both cut-off values together was 75% sensitive and 99% specific for AL
Gong et al^22^	2014	China	Prospective cohort	460	Anterior resection for rectal cancer	161:229	Median 65 (49-72)	35 (7.6%)	pH ≤6.978 POD 3 was independently predictive of the development of clinical AL
Yang et al^23^	2013	China	Retrospective cohort	753	Anterior resection for rectal cancer	452:301	Median 65 (50-74)	57 (7.6%)	pH 6.978 POD 3 was 98.7% sensitive and 94.7% specific for AL
Millan et al^24^	2006	Spain	Prospective cohort	90	Rectal or rectosigmoid resection for cancer of the distal sigmoid colon or rectum	50:40	Mean 66 (38-90)	10 (11.1%) - 4 subclinical, 6 clinical	Patient with a pH <7.28 in the first 24 h postoperatively have 22x more risk of AL

Abbreviations: N, Number; POD, Post-operative Day; AL, Anastomotic Leak.

### pH as a Biomarker for Anastomotic Leak Detection

Nine of 10 studies demonstrated statistically significant lower pH values in anastomoses that had leaked.^18,19,21-27^

**Figure 2.**
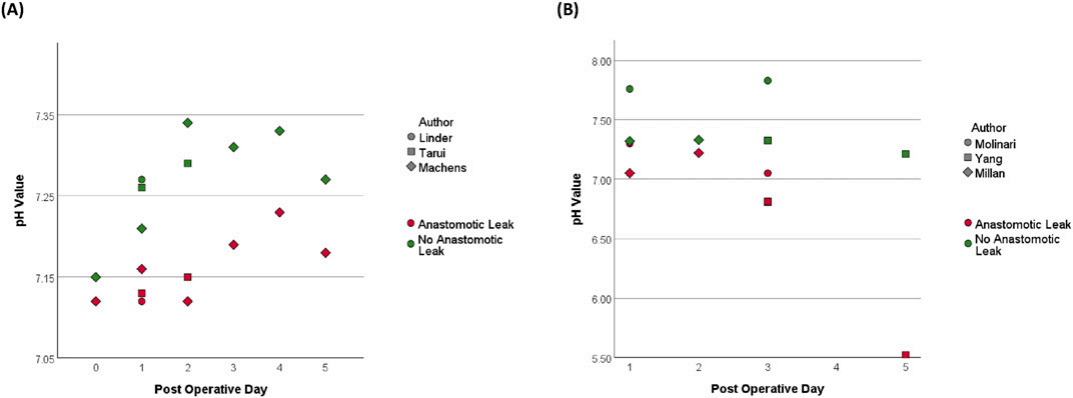
Reported pH values in patients with and without anastomotic leak in studies that report pH values and their post-operative day; (A) upper gastrointestinal, (B) colorectal.

The three studies conducted on upper GI patients following oesophagectomy highlighted the utility of pH monitoring using gastric tonometry in the detection of AL, with pH values summarised in Figure 2A. Linder et al^25^ observed that each surgical step altering the vascular supply to the gastric conduit in 32 patients undergoing oesophagectomy resulted in a detectable decrease in mean pH when compared with the previous step, and a significant increase in mean pH following anastomosis completion (7.15 +/− 0.13 vs 7.21 +/− 0.11, *P* < 0.05). In patients with major AL, defined as Clavien-Dindo^28^ ≥3b, the pH was significantly lower on the first POD compared to those with no AL (7.12 +/− 0.05 vs 7.27 +/− 0.07, *P* = 0.04). This was confirmed by Tarui et al,^26^ who noted significantly lower mean gastric pH levels in patients who experienced AL (13 out of 39 patients), both on the evening of POD 1 (7.13 +/− 0.05 vs 7.26 +/− 0.02, *P* < 0.05) and the morning of POD 2 (7.15 +/− 0.05 vs 7.29 +/− 0.01, *P* < 0.01). They also calculated corrected pH by subtracting gastric pH values from rectal pH values, finding a significant difference from the morning of POD 1 until the morning of POD 2 (*P* < 0.05). However, they observed that while mean rectal pH values increased gradually postoperatively, there was no difference between the two groups. Machens et al^27^ also found that the median pH in six patients with AL was lower immediately following surgery and for the following five days, compared to the six patients with no AL. However, they noted limited clinical benefit in identifying AL ahead of clinical or radiological evidence.

Similarly, in studies focusing on colorectal patients, pH monitoring proved valuable in the detection of AL, with pH values summarised in Figure 2B. Millan et al^24^ utilised tonometry in the stomach and at the anastomosis in 90 patients that had a rectal or rectosigmoid resection. The pH was found to be significantly lower in patients with AL at both the gastric level (7.3 vs 7.37, *P* = 0.027) and anastomotic level (7.05 vs 7.32, *P* = 0.0015) at 24 h postoperatively. However, only anastomotic level pH at 24 h emerged as an independent risk factor for AL on multivariate analysis (*P* = 0.001). Molinari et al^21^ investigated pelvic drain fluid pH in 173 patients undergoing colorectal resection for either cancer or diverticulosis. They identified a significantly lower pH in patients with AL on both POD 1 (7.3 vs 7.76, *P* < 0.001) and POD 3 (7.05 vs 7.83, *P* > 0.001). Furthermore, a retrospective cohort of 753 patients by Yang et al^23^ observed that an early and persistent decline of pH from pelvic drain fluid was indicative of AL, reaching statistical significance from POD 3 through to POD 12 (*P* < 0.001). Contrary to these findings, Ge et al^20^ found no significant difference in pH values on POD 1, 4, or 7 between the AL and non-AL groups from peritoneal drainage following rectal resection in 300 patients (*P* = 0.769).

Seven of the eight human studies reported their method for confirming the presence of AL.^20-24,26,27^ This varied and included a combination of clinical signs (examination findings, systemic signs of infection, discharge from the drain or wound), laboratory tests (raised white blood cell count), radiological diagnosis (computerised tomography (CT), water-soluble contrast enema or swallow), and re-operative findings.

Four studies classified the occurrence of AL, with definitions varying (Table 2). All upper GI studies classified AL as major or minor,^25-27^ with only Linder et al^25^ noting a significant difference in pH between patients with major AL and those without AL on POD 1. Millan et al^24^ classified colorectal AL as clinical vs subclinical, however the combined reporting impedes the comparison of pH between these groups.

**Table 2.** Definitions and pH Analysis of Anastomotic Leak Subgroups.

First author	Year	Origin	Design	Number of participants	Operation(s) performed	Major/Clinical AL definition	Minor/Subclinical AL definition	pH classification analysis
Human Studies - Upper Gastrointestinal								
Linder et al^25^	2017	Sweden	Prospective cohort	32	Oesophagectomy	Clavien Dindo more than 3b	No definition	Patients with major leaks had a lower mean pH on the first postoperative day compared to patients without anastomotic leakage
Tarui et al^26^	1999	Japan	Prospective cohort	39	Oesophagectomy	Discharge from the anastomotic site or gastric tube requiring dressing changes more than twice a day and showed systemic signs of infection	Little discharge and no signs of systemic infection	No significant difference between mean pH values of patients who experienced major vs minor AL
Machens et al^27^	1996	Germany	Prospective cohort	12	Oesophagectomy	Cervical leaks confirmed on exploration of the neck wound due to clinical suspicion from local inflammation aor when air or saliva was found in the cervical drain bag	Identified only in routine contrast studies	The predictive properties of gastric tonometry for early leak detection decreased if only major leaks were considered
Human Studies - Colorectal								
Millan et al^24^	2006	Spain	Prospective cohort	90	Rectal or rectosigmoid resection	Gas, pus or fecal discharge from the drain, pelvic abscess, peritonitis, or discharge of pus per rectum.	Detected by CT or enema	No subgroup analysis

Abbreviation: AL, Anastomotic Leak.

### Method of pH Detection

Three main strategies were used to measure pH in the studies (Table 3). The most frequently used method of pH measurement used in the studies was the analysis of peritoneal drain fluid.^18,20-23^ Four studies used tonometry to monitor intraluminal carbon dioxide, allowing for derivation of intracellular pH using the Henderson-Hasselbalch equation.^24-27^ Among these, two studies included both gastric and rectal tonometry readings,^24,26^ with Tarui et al^26^ additionally calculating corrected pH by subtracting the rectal pH value from the gastric pH value. Only one animal study utilised colonic intramural pH monitoring.^19^

**Table 3.** Method of pH Determination and Monitoring.

First author	Year	Origin	Design	Number of participants	Operation(s) performed	Method of pH detection	Calibration method	Temperature
Animal Studies								
Huynh et al^18^	2023	Canada	In vivo models (pigs)	2 pigs	Open abdomen leak (gastrotomy)	Extraluminal pH using polyaniline (PANI)-based sensor (FluidAI Medical, Kitchener, Canada) connected inline to peritoneal drain	Set of standard pH buffers (pH 4.0, 7.0 and 10.0) (HANNA Instruments, Quebec, Canada). Linear calibration curves for sensor output vs pH obtained using buffers.	On-chip thermistor to allow for temperature-correction of pH measurements
					Closed abdomen leak (needle aspiration of gastric fluid followed by open leak)			
Senagore et al^19^	1990	USA	In vivo models (pigs)	35 pigs	Colorectal resection at the sacral promontory with division of mesentery 10 cm proximal and distal to induce ischaemic insult	Intramural pH probes (Khuri tissue pH probes, Vascular Technology Inc., North Chelmsford, MA) at 1 cm proximal to, at, and 1 cm distal to the anastomosis	Not stated	Thermistor probe to allow for determination of local temperature for Nernst equation
Human Studies - Upper Gastrointestinal								
Linder et al^25^	2017	Sweden	Prospective cohort	32	Oesophagectomy	Gastric tonometry catheter with recording by Datex-Ohmeda S5 Tonometry Module (M-TONO, Datex- Ohmeda, Helsinki, Finland) and arterial pH	Initial calibration time of 30 min recommended by the manufacturer for washout of air in the balloon and catheter	Not stated
Tarui et al^26^	1999	Japan	Prospective cohort	39	Oesophagectomy	Gastric and rectal tonometry (Trip, Tonometrics, Worcester, MA, USA)	2.5 mL 0.9% saline interted into tonometer balloon for 30-60 min	Not stated
						Corrected pH = Gastric pH - Rectal pH		
Machens et al^27^	1996	Germany	Prospective cohort	12	Oesophagectomy	Gastric tonometry (Tonometrics. Worcester, MA, USA) 2 cm distal to cervical oesophagogastric anastomosis	Not stated	Not stated
Human Studies - Colorectal								
Ge et al^20^	2021	China	Prospective cohort	300	Rectal resection	Extraluminal pH using peritoneal drain around anastomosis, tested using a fully automated urine analyser (AUTION MAX AX-4030, ARKRAY Corporation, Japan)	Not stated	Not stated
Molinari et al^21^	2019	Italy	Prospective cohort	173	Right hemicolectomy, left hemicolectomy, lower anterior resection	Extraluminal pH using pelvic drain, 10 mL sample taken for pH and chemical analysis	Not stated	Not stated
Gong et al^22^	2014	China	Prospective cohort	460	Anterior resection	Extraluminal pH using pelvic drain, method for pH testing not stated	Not stated	Not stated
Yang et al^23^	2013	China	Retrospective cohort	753	Anterior resection	Extraluminal pH using pelvic drain, tested using ph meter (pp-15, Sartorious Ltd., Germany)	Not stated	pH value determined at 25°c
Millan et al^24^	2006	Spain	Prospective cohort	90	Rectal or rectosigmoid resection	Gastric and anastomotic tonometry (Trip, Tonometrics, Worcester, MA, USA)	2.5 mL 0.9% saline interted into tonometer balloon for 60 min	Not stated

Four studies described the methods used to calibrate pH measurements.^18,24-26^ Three of these studies utilised tonometric devices calibrated with saline solution,^24-26^ and one study used a set of pH buffers to calibrate a pH sensor.^18^ Temperature considerations for pH measurements were noted in three studies, with two animal studies using thermistors to enable temperature-correction of pH measurements,^18,19^ while Yang et al^23^ determined the pH value at 25°c using a pH meter.

### Frequency and Timing of pH Monitoring for Anastomotic Leak Detection

The included studies exhibited variability in the frequency of pH readings and the subsequent timing for AL detection (Table 4). All human studies utilised non-continuous monitoring, with a median postoperative reading interval of 24 h (12 h - 72 h).

**Table 4.** Frequency of pH Monitoring for Anatomotic Leak Detection.

First author	Year	Origin	Design	Number of participants	Operation(s) performed	Frequency of pH monitoring	POD for AL detection with pH	POD for AL detection without pH
Animal Studies								
Huynh et al^18^	2023	Canada	In vivo models (pigs)	2 pigs	Open abdomen leak (gastrotomy)	Continuous	Within 10 min of leak induction	–
					Closed abdomen leak (needle aspiration of gastric fluid followed by open leak)			
Senagore et al^19^	1990	USA	In vivo models (pigs)	35 pigs	Colorectal resection at the sacral promontory with division of mesentery 10 cm proximal and distal to induce ischaemic insult	POD 5, 11, 21, and 60	Not stated	–
Human Studies - Upper Gastrointestinal								
Linder et al^25^	2017	Sweden	Prospective cohort	32	Oesophagectomy	Intraoperatively 11-19 min after each 6 key steps, 2 h postoperatively, morning of POD1 (median 18 h, range 15-23 h)	Significant difference on POD 1	Not stated
Tarui et al^26^	1999	Japan	Prospective cohort	39	Oesophagectomy	Intraoperatively when gastric tube complete, end of the operation, 2 h post-operatively, morning and evening POD 1, morning POD 2.	Significant difference on evening POD 1 and morning POD 2	Anastomotic insufficiency was clinically observed on POD 4
Machens et al^27^	1996	Germany	Prospective cohort	12	Oesophagectomy	After anastomotic completion and daily thereafter at 09:00 h	Median pH lower immediately after surgery and on the following 5 days	Major leaks manifested by local inflammation at the anastomotic site (two patients on days
								3 and 10) or by air (one patient on day 1) or saliva (one patient on day 10) in the cervical drain bag
Human Studies - Colorectal								
Ge et al^20^	2021	China	Prospective cohort	300	Rectal resection	POD 1, 4, and 7	Not detected - No difference in groups	Not stated
Molinari et al^21^	2019	Italy	Prospective cohort	173	Right hemicolectomy, left hemicolectomy, lower anterior resection	POD 1 and 3	Significant difference on POD 1 and 3	AL became clinically relevant between POD 4 and 8 (mean POD 4)
Gong et al^22^	2014	China	Prospective cohort	460	Anterior resection	POD 3 (from Yang et al)	Significant difference on POD 3	AL diagnosis made between POD 6 and 12 (mean POD 8)
Yang et al^23^	2013	China	Retrospective cohort	753	Anterior resection	Daily for 12 days	Significant difference from POD 3 to 12	AL diagnosis made between POD 6 and 12 (mean POD 8)
Millan et al^24^	2006	Spain	Prospective cohort	90	Rectal or rectosigmoid resection	Early postoperative period (first 24 h) and repeated on second postoperative day (first 48 h)	Significant difference at anastomotic level at in first 24 h	Not stated

Abbreviations: POD, Post-operative Day; AL, Anastomotic Leak.

All three studies involving upper GI patients undergoing oesophagectomy initiated pH monitoring intraoperatively using tonometry.^25-27^ Among these, two studies identified a significantly lower pH in patients with AL on POD 1.^25,26^ The third study by Machens et al^27^ identified a lower median pH immediately after surgery and on the following 5 days, noting that pH values in AL after POD 3 were generally lower than those within the first 3 days postoperatively. Two additional studies focusing on colorectal patients also identified a significantly lower pH in patients with AL on POD 1.^21,24^ However, Yang et al^23^ found no significant difference in pH on POD 1 and 2. They observed a sharp decline in mean pH on POD 3, reaching a diagnostic threshold (6.811 in AL vs 7.325 in non-AL, *P* < 0.001) that remained significant until POD 12, with the lowest mean pH reading on POD 5 in those with AL (pH 5.522). Gong et al^22^ confirmed these findings by Yang et al,^23^ identifying that the pH value less than or equal to 6.978 in pelvic drain fluid on POD 3 was significantly associated with the development of clinical AL (*P* < 0.001).

An animal study by Senagore et al^19^ utilised the longest postoperative pH monitoring period of 60 days. They identified a 70% anastomotic complication rate overall, however the timing of AL detection using pH was not reported.

Continuous monitoring of drain fluid pH using inline sensors was utilised in one animal study involving simulated leaks in two pigs.^18^ The sensors successfully detected the resulting local changes in drain fluid pH within 10 min of leak induction.

Five papers reported the time that AL became clinically observed.^21-23,26,27^ Although most of these studies report a shorter time for AL detection with pH, there are no statistical comparisons in diagnosis time using pH vs the reported methods for clinical detection.

### Diagnostic Test Accuracy of pH for Anastomotic Leak Detection

Five of the included studies specified a pH cut-off value for the diagnosis of AL (Table 5).^19,21,23,24,27^ Molinari et al^21^ identified that drain fluid pH of <7.53 on POD 1 and <7.21 on POD 3 were both independent predictors of AL. They concluded that pH on POD 1 was useful for selecting patients who will not develop AL (positive predictive value (PPV) 24.1%, negative predictive value (NPV) 99.1%) while POD 3 may identify those requiring more careful management (PPV 70.59%, NPV 97.44%), and by combining these values they were able to increase the diagnostic performance of the pH (PPV 85%, NPV 97.4%). The lowest identified cut-off value was 6.978 at POD 3,^23^ this yielded a sensitivity and specificity of 98.7% and 94.7% respectively. This cut-off value was explored further by Gong et al^22^ who identified this value on POD 3 as an independent predictive factor for the development of clinical AL (*P* < 0.001, OR 283.71, 95% CI 25.54 - 3152.11). However, the wide range in confidence intervals suggests substantial variability in the estimated odds for predicting AL.

**Table 5.** pH Cut-Off Values and Diagnostic Test Accuracy Data. POD = Post-operative Day. ROC = Receiver Operator Characteristics.

First author	Year	Origin	Design	Number of participants	Operation(s) performed	pH cut-off	Timing	ROC curve AUC	Sensitivity	Specificity	Positive Predictive Value	Negative Predictive Value	Accuracy
Machens et al^27^	1996	Germany	Prospective cohort	12	Oesophagectomy	7.2	POD 2	–	80%	67%	71%	80%	75%
Millan et al^24^	2006	Spain	Prospective cohort	90	Rectal or rectosigmoid resection	7.28	24 h	0.861	90.0%	71.3%	28.1%	98.3%	73.3%
Molinari et al^21^	2019	Italy	Prospective cohort	173	Right hemicolectomy, left hemicolectomy, lower anterior resection	7.53	POD 1	0.80	93.75%	70%	24.1%	99.1%	–
						7.21	POD 3	0.86	75%	97%	70.59%	97.44%	–
						Both 7.53 POD 1 and 7.21 POD 3		0.97	75%	99%	85%	97.4%	–
Senagore et al^19^	1990	USA	In vivo models (pigs)	35 pigs	Colorectal resection at the sacral promontory with division of mesentery 10 cm proximal and distal to induce ischaemic insult	7.0	Not stated	–	–	–	100%	89%	93%
Yang et al^23^	2013	China	Retrospective cohort	753	Anterior resection	6.978	POD 3	–	98.7%	94.7%	–	–	–

## Discussion

This review presents a comprehensive analysis of pre-clinical and clinical studies focusing on pH monitoring for the detection of AL. The included studies demonstrate the potential of pH monitoring as a tool for early AL detection, with lower pH values consistently observed in patients with AL across various anatomical locations and postoperative periods. Diverse pH measuring and monitoring techniques were employed, with gastric tonometry predominantly utilised in upper GI patients and peritoneal drain fluid analysis for colorectal patients. Variability in the frequency of pH readings and timing for AL detection was observed, and further research is required to determine diagnostic accuracy.

The typical pH of peritoneal fluid ranges between 7.5 and 8.0, with significant buffering capacity.^29^ The observed decrease in postoperative pH in patients with AL can be attributed to a series of microenvironment changes, beginning with reduced tissue perfusion.^21,30^ Although the pathogenesis of AL is not fully understood, it is recognised that leaks seem to be a multifactorial failure of wound healing, rather than solely due to technical error.^12^ Additionally, surgical trauma decreases the pH of inflammatory exudates,^23,31^ which may also account for the reduced post-operative pH values in patients without AL. Furthermore, patients undergoing surgery might experience systemic reductions in pH due to factors such as pre-operative fasting, hypovolaemia, and peripheral shutdown.^32^ This may also explain the finding by Millan et al^24^ that both the gastric and anastomotic pH were lower at 24 h postoperatively in colorectal AL.

Current diagnostic methods generally detect AL between day 5 and day 8 post-operatively, however, they exhibit variable sensitivity and specificity, along with logistical constraints that may delay timely intervention.^33^ The studies included in this review demonstrate that pH monitoring may detect alterations in pH levels associated with AL as early as the first post-operative day in both upper GI and colorectal patients, potentially facilitating prompt intervention and mitigating the severity of AL-related complications. However, before pH can be considered a useful biomarker for the early detection of AL, several issues need further clarification. More research is needed to establish the accuracy and predictive values of pH monitoring, to determine the cut-off pH values that differentiate AL from normal tissue inflammation and healing, and to determine the biological variability in defined patient populations.

Integrating pH monitoring into routine postoperative care protocols could offer an additional valuable tool for multifactoral risk stratification. This would allow clinicians to tailor interventions including closer monitoring, further investigations, early initiation of nutrition^34^ and antibiotic therapy, non-surgical interventions including percutaneous drainage^35^ or endoscopic management,^36,37^ and timely return to the operating theatre when necessary.

However, the potential influence of perioperative factors on pH monitoring needs consideration. While the use of combined mechanical and oral antibiotic bowel preparation has demonstrated a reduced risk of AL,^38-40^ there was inconsistent reporting and methods regarding preoperative preparation across studies in this review. Furthermore, no analysis was conducted to assess the potential impact of these practices on pH values. In patients undergoing emergency surgery, factors such as pre-existing peritonitis and severe systemic inflammation may alter pH, in addition to potentially confounding patient outcomes. Moreover, additional factors such as pre-operative carbohydrate loading, resumption of oral intake, and influence of postoperative complications including ileus and surgical site infection should be evaluated and efforts made to standardise potential confounders.

Enhanced Recovery After Surgery (ERAS) protocols have been effective in reducing postoperative complication rates and hospital length of stay.^41,42^ In ERAS protocols, routine placement of intra-abdominal drains after colorectal surgery is discouraged due to evidence suggesting no improvement and potential worsening of postoperative outcomes.^43,44^ Historically, prophylactic drains were thought to aid with the evacuation of perianastomotic fluid collections and enable early AL diagnosis through faeces or pus content in the drain.^45^ However, this is not supported in the literature with a recent meta-analysis of RCTs identifying that routine prophylactic drain placement after colorectal anastomosis did not prevent clinical or radiological AL, or decrease the clinical sequelae of AL-related complications.^46^ Furthermore, multiple studies report an increased rate of drain-related complications including wound infection in patients who had abdominal drains inserted following GI surgery.^47-49^ Despite this, the use of drains is practised selectively according to surgeon preference^50^ and the potential impact of drain fluid pH analysis for early AL detection remains to be fully evaluated. The implementation of pH monitoring requires careful consideration, balancing the potential benefits with the associated logistical and cost implications which conflict with the goal of streamlining postoperative care.

### Limitations

A narrative synthesis was performed to summarise the diverse range of identified studies in a structured manner. However, due to the wide heterogeneity of the literature included in this review, we were unable to conduct a quantitative comparison outcome measure. Particularly, there was a large variation in reading frequency and pH cut-off values for AL detection, largely due to the limited evidence currently available, and therefore we are unable to give clear evidence-based guidance. Furthermore, while the included studies demonstrate the potential utility of pH monitoring in identifying AL, further research is required to evaluate its impact on clinical management and patient outcomes.

Future work in this field should focus on establishing standardisation of pH monitoring for AL detection including frequency of monitoring and methods of measurement to ensure consistency and comparability across studies and to facilitate the integration into clinical practice. Given the varying use of intra-abdominal drains, future studies should also explore patient and surgeon acceptance which is a crucial factor influencing adoption in clinical practice. All human studies were single-centre, with one retrospective cohort and the remainder prospective cohort designs. The conduct of high-quality well-designed diagnostic test accuracy studies and randomised trials would further elucidate the accuracy and reliability of pH monitoring in AL to allow for the assessment of the clinical utility in guiding patient management decisions.

## Conclusion

These findings collectively highlight the potential role of pH monitoring as a tool for early anastomotic leak detection following gastrointestinal surgery. The reported sensitivities range from 75.0% to 98.7% and specificities from 70.0% to 99.0%, suggesting its potential as an early warning system for clinicians to tailor their management strategies to allow for timely intervention and improved patient outcomes. Future recommendations for research include examining the feasibility and cost-effectiveness of routine pH assessment as part of postoperative care, the validation of emerging pH sensor technologies, and the clinical use of real-time continuous monitoring to provide a dynamic and instantaneous understanding of pH alterations associated with anastomotic leaks.

## Appendix 1 Database Search Strategy

**Table:**

Search Number	Keyword
1	Gastr*
2	Colo*
3	Rect*
4	Esophag*
5	Oesophag*
6	Anastomo*
7	Heal*
8	Leak*
9	Fail*
10	Insufficien*
11	pH
12	Acid*
13	Impedance
14	1 OR 2 OR 3 OR 4 OR 5
15	7 OR 8 OR 9 OR 10
16	11 OR 12
17	13 OR 16
18	6 AND 14 AND 15 AND 17
19	Limit 18 to English language
